# Retinoblastoma cells activate the AKT pathway and are vulnerable to the PI3K/mTOR inhibitor NVP-BEZ235

**DOI:** 10.18632/oncotarget.16970

**Published:** 2017-04-08

**Authors:** Chencheng Xie, Matthew J. Freeman, Huarui Lu, Xiaohong Wang, Colleen L. Forster, Aaron L. Sarver, Timothy C. Hallstrom

**Affiliations:** ^1^ Department of Pediatrics, University of Minnesota, Minneapolis, MN, USA; ^2^ BioNet, Academic Health Center, University of Minnesota, Minneapolis, MN, USA; ^3^ Masonic Cancer Center, University of Minnesota, Minneapolis, MN, USA

**Keywords:** retinoblastoma, AKT, NVP-BEZ235, carboplatin, topotecan

## Abstract

Retinoblastoma is a pediatric cancer of the retina most often caused by inactivation of the retinoblastoma (*RB1)* tumor suppressor gene. We previously showed that *Rb1* loss cooperates with either co-activating the phosphatidylinositol 3-kinase (PI3K)/AKT pathway, or co-deleting *Pten*, to initiate retinoblastoma tumors in mice. The objectives of this study were to determine if the AKT pathway is activated in human retinoblastomas and the extent that anti-PI3K therapy induces apoptosis in retinoblastoma cells, alone or in combination with the DNA damaging drugs carboplatin and topotecan. Serial sections from human retinoblastoma tissue microarrays containing 27 tumors were stained with antibodies specific to p-AKT, Ki-67, forkhead box O1 (p-FOXO1), and ribosomal protein S6 (p-S6) using immunohistochemistry and each tumor sample scored for intensity. Human retinoblastoma tumors displayed significant correlation between p-AKT intensity with highly proliferative tumors (*p* = 0.008) that were also highly positive for p-FOXO1 (*p* = 0.002). Treatment with BEZ235, a dual PI3K/mTOR inhibitor, reduced phosphorylation levels of the AKT targets p-FOXO and p-S6 and effectively induced apoptosis the Y79 and Weri-1 human retinoblastoma cell lines and *in vivo* in our retinoblastoma mouse model. Long-term treatment with BEZ235 *in vivo* using our retinoblastoma-bearing mice induced apoptosis but did not significantly extend the lifespan of the mice. We then co-administered BEZ235 with topotecan and carboplatin chemotherapeutics *in vivo*, which more effectively induced apoptosis of retinoblastoma, but not normal retinal cells than either treatment alone. Our study has increased the variety of potentially effective targeted treatments that can be considered for human retinoblastoma.

## INTRODUCTION

Retinoblastoma is a pediatric tumor of the retina resulting from biallelic inactivation of the retinoblastoma tumor suppressor gene *RB1*. Early detection and new treatment options have improved survival of retinoblastoma patients. Improved therapies are still needed for patients with bilateral disease, however, who often lose vision through enucleation. Treatment options for retinoblastoma include local or systemic chemotherapy, external-beam or plaque radiation therapy, cryo- and thermo- therapy, or surgery to enucleate one or both eyes. Carboplatin is a standard chemotherapeutic agent for retinoblastoma treatment and has been used alone or in combination with other drugs such as vincristine or etoposide. Systematic testing of pairs of chemotherapeutic drugs on retinoblastoma cells revealed that topotecan and carboplatin were more effective halting retinoblastoma progression when combined than other DNA-damaging agents alone or in combination [1, 2]. However, combined treatment with carboplatin and topotecan causes dose-limiting myelosuppression in humans with solid tumors [3]. Delivering carboplatin via subconjunctival injection can bypass myelo-suppressive side effects and maximize therapy directly at the tumor [4]. It also more effectively reaches tumor vitreal seeds, which are difficult to treat. This has led to the development of chemotherapy infusion delivered through the ophthalmic artery, because local administration can help in cases where systemic treatment causes significant side-effects. Ophthalmic arterial delivery of these agents was found to be effective in humans [5].

Loss of the *RB1* tumor suppressor gene is the primary driving mutation in human retinoblastoma [6, 7]. Loss of RB activates the E2F transcription factors, leading to gene expression that controls a feed-forward proliferative and apoptotic response [8–13]. Alterations in several members of the p53 pathway, such as ARF, MDM2 and MDMX have been implicated in suppressing apoptosis control in developing retinoblastoma tumors [7, 14–18]. Work from our lab has also demonstrated that inactivation of phosphatase and tensin homolog (PTEN), or constitutive activation of PI3K or AKT, suppresses Rb/E2F driven apoptosis in the retina and contributes to retinoblastoma formation in mice [19]. *PTEN* is a tumor suppressor gene encoding a lipid phosphatase that opposes the activity of PI3K kinases, and both genes are frequently altered in diverse human cancers. PTEN loss or PI3K activation leads to phosphorylation and activation of AKT (P-AKT), a serine/threonine kinase that directly phosphorylates a wide variety of targets to control cell survival, protein synthesis and glucose metabolism [20]. The mechanistic target of rapamycin (mTOR) is also downstream of PI3K/AKT and is comprised of distinct complexes which can phosphorylate ribosomal protein S6 kinase B1 (RPS6KB1). AKT directly phosphorylates FOXO proteins, a family of transcription factors that control expression of genes involved with cell death, DNA repair, metabolism and oxidative stress detoxification [21]. In our mouse retinoblastoma model, an E2F1/FOXO pro-apoptotic transcriptional complex, which is active following disruption of RB and p107, induces widespread apoptosis that results in loss of most of the retinal. This pro-apoptotic complex can be disabled by AKT phosphorylation of FOXOs, or by introducing a dominant-negative FOXO *in vivo* in the retina, thereby inducing rather than suppressing tumorigenesis.

The goals of this current study were two-fold. The first was to determine if the p-AKT/p-FOXO pathway is altered in human retinoblastoma. The second was to utilize our mouse retinoblastoma model as a pre-clinical model to test the efficacy of PI3K/mTOR inhibition, alone or in combination therapy with chemotherapeutic agents. We observed p-Akt activation in many human retinoblastoma specimens. p-AKT staining was significantly correlated with Ki-67 positive regions and was predominantly associated with regions that also stained positive for p-FOXO1. Co-activation of Akt and FOXO1 phosphorylation is also a common feature in proliferating human retinoblastoma tumor cells. *ΔPten*-driven tumors depend on signaling through unchecked PI3K activity [22–24]. Thus, our retinoblastoma mouse model is amenable to preclinical investigation of the efficacy of PI3K inhibition and combination therapy. NVP-BEZ235 (hereafter referred to as BEZ235) is a PI3K/mTOR dual inhibitor being tested as an anti-cancer therapeutic in various clinical trials. BEZ235 induced apoptosis of retinoblastoma cells *in vitro* and *in vivo*. We observed that the combined treatment of BEZ235 with topotecan and carboplatin caused retinoblastoma cells to undergo apoptosis *in vivo* much more effectively than either topotecan/carboplatin or BEZ235 treatment.

## RESULTS

**Figure 1:**
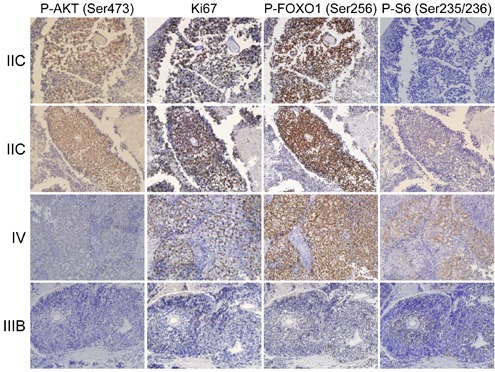
The Akt/FoxO pathway is active in human retinoblastoma Human tissue microarrays (TMAs) containing 27 retinoblastoma tumors were stained with antibodies specific to p-AKT, Ki-67, p-FOXO1, and p-S6. Tumor stage is listed to the left of images. Serial sections were stained so the relative intensity could be compared, and representative images are shown for a range of p-Akt staining intensities.

It is not known if the AKT pathway is activated in human retinoblastoma. We performed immunohistochemistry (IHC) on human retinoblastoma samples to determine if the Akt pathway is phosphorylated and activated (Ser473 p-AKT) in these tumors. In total, 27 retinoblastomas were stained on commercial tissue microarray (TMA) for these analyses. We observed positive p-AKT staining of many retinoblastomas, indicating that a fraction of these tumors activated the p-AKT pathway. Representative images are shown for a range of p-Akt staining intensities (Figure 1). Serially sectioned adjacent tissues were also stained with α-Ki-67 antisera to identify proliferating regions and with α-p-FOXO1 (Ser256) and α-p-S6 (Ser235/236) as markers of phospho-Akt activity. Importantly, staining of all samples was conducted simultaneously to minimize sample to sample variation. Staining intensities were assigned a relative score from 0 (no staining) to 4 (most intense staining) across a total of 27 retinoblastomas for each set of IHC experiments. Shown adjacent to p-Akt staining are the IHC for Ki-67, p-FOXO1, and p-S6 on serially stained sections. The relative staining intensity of p-Akt, Ki-67, p-FoxO1, and p-S6 was scored for each individual retinoblastoma ranging from 0 (no staining) to 4 (most intense staining) (Table S1). We converted staining results into 2 × 2 contingency tables for Fisher's exact tests to determine if staining intensities significantly correlate. The total numbers of samples in each row and column are displayed. For the first test (Table 1A), rows were separated based on p-Akt intensity scores of pAkt >= 2 or pAkt < 2. Columns were separated into relative staining intensities for Ki-67 < 3 or Ki-67 >= 3. Fisher's exact test computed a two-tailed P-value of 0.008 for this contingency table, indicating that highly proliferative human retinoblastomas displayed high levels of p-Akt activation.

**Table 1:** p-AKT staining intensity correlates with Ki-67^+^ and p-FOXO1^+^ tumor staining

**A**		**High Ki-67**	**Low Ki-67**	**Row Totals**
	High p-AKT	11	8	19
	Low p-AKT	0	8	8
	Column Totals	11	16	*p* = 0.008
**B**		**High p-FOXO1**	**Low p-FOXO1**	**Row Totals**
	High p-AKT	6	0	6
	Low p-AKT	5	16	21
	Column Totals	11	16	p=0.002

The relative staining intensity of p-Akt, Ki-67, p-FoxO1, and p-S6 was scored for each individual retinoblastoma. Comparisons between p-AKT and Ki-67 (A) or p-AKT and pFOXO1 (B) were converted to a 2 × 2 contingency tables. A. Tumors were separated based on intensity scores of p-AKT >= 2 (High) or p-AKT < 2 (Low) and Ki-67 >= 3 (High) or Ki-67 < 3 (Low). B. Tumors were separated based on intensity scores of p-AKT >= 3 (High) or p-AKT < 3 (Low) and pFOXO1 = 4 (High) or pFOXO1 <= 4 (Low). Total numbers of tumors in each row are displayed. Fisher's exact test was used to calculate two-tailed P-values for the contingency table comparisons.

We further analyzed the correlation between p-AKT and p-FOXO1 activation in human retinoblastoma. Because Akt is known to directly phosphorylate FOXO1, we determined if FOXO1 phosphorylation (p-FOXO1) is high in tumors that also stained highly positive for activated Akt (p-AKT). We converted p-Akt and p-FOXO1 staining results into 2 × 2 contingency tables for Fisher's exact tests to determine if staining intensities significantly correlate. For this test (Table 1B), rows were separated based on p-Akt intensity scores of pAkt >= 3 or pAkt < 3. Columns were separated into relative staining intensities for p-FOXO1 <= 3 or p-FOXO1 = 4. Fisher's exact test computed a two-tailed P-value of 0.002 for this contingency table, indicating that human retinoblastomas with high levels of p-Akt are also highly positive for p-FOXO1. AKT activates the mechanistic target of rapamycin (mTOR) which in turn phosphorylates the S6 ribosomal kinase. We assessed p-S6 levels by IHC and observed p-S6 staining in a subset of p-AKT positive tumor regions, but Fisher's exact test did not calculate any significant correlation between p-Akt and p-S6 levels in these tumors. We conclude that the AKT signaling pathway is activated in human retinoblastomas and significantly correlates with p-FOXO1 and Ki-67 staining in these tumors.

**Figure 2:**
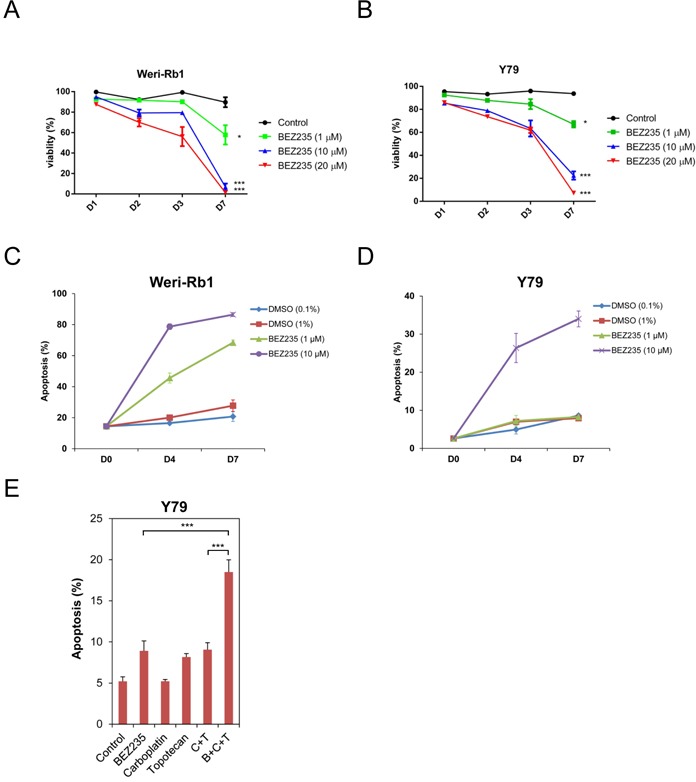
BEZ235 inhibits the growth of human retinoblastoma cell lines **A**. Weri-Rb1 and **B**. Y79 cells were grown in culture, treated with vehicle (control) or with BEZ235 at the listed concentrations. Viability of cells was measured in triplicate by trypan blue dye exclusion at 1-, 2-, 3-, and 7-day time points. Results are graphed as the percentage of viable cells divided by total cells at each time point (% viability). **C**. Weri-Rb1 and **D**. Y79 cells were treated with low and high doses of DMSO (control) or BEZ235 at the concentrations listed. Cells were harvested for apoptosis analysis measuring active, cleaved caspase-3 by flow cytometry. **E**. Y79 cells were treated with control, BEZ235 (1 μM), carboplatin (0.4 μM), topotecan (0.003 μM), carboplatin (0.4 μM) + topotecan (0.003 μM) (C+T), or BEZ235 (1 μM) + carboplatin (0.4 μM) + topotecan (0.003 μM) (B+C+T). Cells were harvested 6 days after treatment and the percent apoptotic cells were measured using flow cytometry targeting active caspase-3. *, p < 0.05; ***, p < 0.001. Error bars represent standard deviation from the mean.

We next tested if two human retinoblastoma cell lines, Weri-Rb1 (Figure 2A, hereafter referred to as Weri1) and Y79 Figure 2B), were susceptible to BEZ235-mediated growth inhibition or apoptosis. These cells were grown with vehicle or increasing amounts of BEZ235. The viability of treated cells were measured at 1-day (D1), 2-day (D2), 3-day (D3) and 7-day (D7) time points and displayed as graphs in Figure 2. Viability of both cell lines was significantly reduced by treatment with 1 μM (p < 0.05), and by 10 μM and 20 μM (each p < 0.005) by the 7-day time point compared to control cells. We determined if this reduction in viability observed in cultured cells is due to induction of apoptosis by measuring active caspase-3 levels by flow cytometry. Weri1 (Figure 2C) and Y79 (Figure 2D) cells were grown with DMSO vehicle or BEZ235 (1 & 10 μM), and harvested immediately (D0), or after 4 (D4) or 7 (D7) days of treatment for flow cytometry and graphed as the percent apoptotic cells. Both Weri1 and Y79 cells underwent apoptosis with 10 μM BEZ235, although Weri1 cells were particularly susceptible, with over 80% of cells having undergone apoptosis by 7-days of treatment. In comparison, ~34% of Y79 cells committed apoptosis under the same conditions. Similarly, 1 μM BEZ235 did not cause significant apoptosis in Y79 cells, but this treatment led to apoptosis of 45% of cells at day 4 and 68% at day 7. We further explored the vulnerability of the more apoptotic resistant Y79 cells by co-treating them with BEZ235 and the chemotherapeutics carboplatin and topotecan. Solo treatment with BEZ235 (1 μM), topotecan (0.003 μM), or combined topotecan (0.003 μM)/carboplatin (0.4 μM) treatment caused a modest increase in apoptosis compared to control treatment (Figure 2E). Carboplatin alone was ineffectual at the 0.4 μM concentration used. Combined treatment with all three at the stated concentrations significantly increased the number of apoptotic cells over BEZ235 alone or carboplatin/topotecan combination.

**Figure 3:**
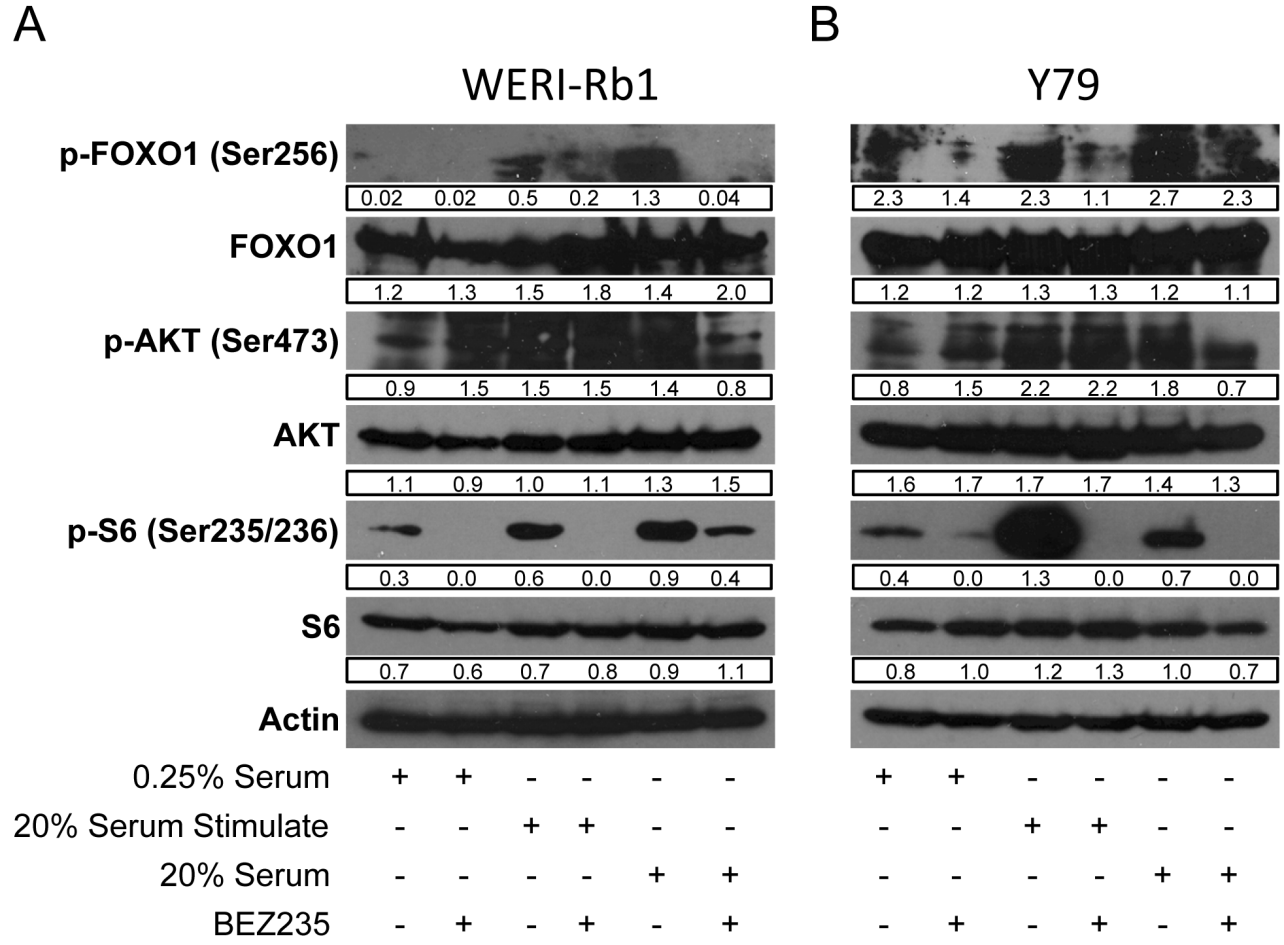
BEZ235 inhibits phosphorylation of AKT targets in human retinoblastoma cell lines **A**. WERI-Rb1 and **B**. Y79 cell lines were grown in media containing 0.25% serum, in 0.25% serum before stimulating with 20% serum for an hour, or grown continuously in 20% serum. Vehicle or BEZ235 (50 μM) was added one hour prior to harvesting cells for protein isolation. Proteins extracts were analyzed by immunoblotting for levels of p-FOXO1 (Ser256), FOXO1, p-AKT (Ser473), AKT, p-S6 (Ser235/236), S6, and ACTIN. Band densities were quantified using ImageJ and reported under each band. Numbers are only comparable to each other within the same gel.

Serum withdrawal and addition affects PI3K and AKT activation and signaling in cultured cells [9, 11]. We grew Weri1 (Figure 3A) and Y79 (Figure 3B) cells under three different conditions: 0.25% serum deprivation, in 0.25% serum before stimulating with 20% serum for one hour, or grown continuously in 20% serum. Cells were further treated with vehicle or BEZ235 to determine how serum and PI3K/Akt/mTOR coordinate activation of phosphorylated AKT (p-AKT), and the downstream AKT phospho-targets p-FOXO1 and p-S6 to better understand how these pathways are regulated in these cell lines. Vehicle or BEZ235 (50 μM) was added one hour prior to harvesting cells for protein isolation. Proteins extracts were isolated and tested by immunoblotting for levels of p-FOXO1 (Ser256), FOXO1, p-AKT (Ser473), AKT, p-S6 (Ser235/236), S6, and ACTIN.

FOXO1 is regulated differently in Weri1 (3A) than Y79 (3B) cells. P-FOXO1 is present in serum deprived Y79 but not Weri1 cells. FOXO1 phosphorylation is stimulated by brief addition of serum for one hour in Weri1, but not Y79 cells. P-FOXO1 was further elevated in Weri1 cells grown in 20% serum, whereas only a modest boost was observed in asynchronously growing Y79 cells. These findings indicate that FOXO1 phosphorylation is less dependent upon addition of growth factors in Y79 compared with Weri1 cells. Treatment with BEZ235 also affects p-FOXO1 differently, almost completely inhibiting its phosphorylation in asynchronously grown Weri1 cells but having limited effect in Y79 cells. In contrast, p-AKT is present in both Y79 and Weri1 cells, even in 0.25% serum which is typically insufficient to activate this pathway in other cell types [11]. p-AKT levels modestly increase following brief or long-term serum stimulation in both Weri1 (~1.5-fold) and Y79 (~2-fold) lines. BEZ235 treatment in 0.25% serum deprived Weri1 and Y79 cells led to a 50% increase in p-AKT levels, but the reason for this increase is unknown. Treatment with BEZ235 did not affect p-AKT levels in either Weri1 or Y79 cells, suggesting the possibility that AKT is targeted for phosphorylation independently of PI3K activity following stimulation with growth factors. P-AKT was reduced by ~50% in both cell lines, to levels observed in 0.25% serum grown cells, upon BEZ235 treatment in asynchronously growing cells. P-S6 appears constitutively phosphorylated in both cell lines in 0.25% serum levels, and its levels rise significantly in both lines, although much more dramatically in Y79 cells, following treatment with 20% serum. BEZ235 treatment significantly reduced p-S6 levels under each of the serum treatments, except in Weri1 cells that retain significant p-S6 phosphorylation in asynchronously grown, BEZ235 treated cells. The levels of FOXO1, AKT1, S6 and ACTIN proteins did not change by any of the treatments. Y79 and Weri1 cells show nuanced differences in their regulation of these pathways, with Y79 appearing to more strongly activate p-FOXO1 levels independent of growth factor addition. Also, Y79 cells more strongly induced p-S6 levels following stimulation, but Weri1 cells failed to fully inhibit p-S6 by BEZ235 in asynchronously growing cells. It is not yet clear why p-AKT remains high in both lines following serum stimulation combined with BEZ235 inhibition, or why p-FOXO1, a direct Akt phospho-target, levels drop under these conditions without a correlating drop in p-AKT levels.

**Figure 4:**
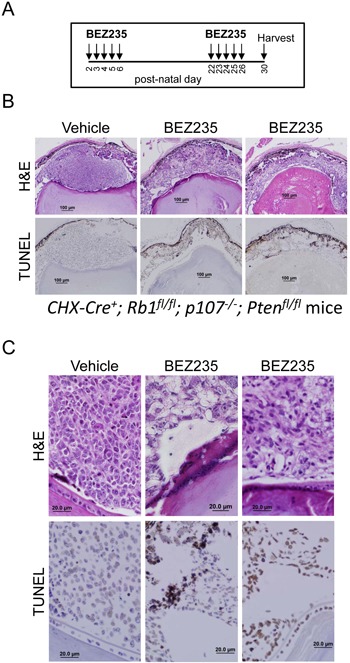
BEZ235 induces apoptosis in murine retinoblastoma cells **A**. Schema for BEZ235 delivery to *CHX-Cre^+^; Rb1^fl/fl^; p107^−/−^; Pten^fl/fl^* mice. These mice develop rapid (<30 day) bilateral retinoblastoma tumors. Vehicle or BEZ235 was delivered by oral gavage on post-natal days 2-6 and 22-26 and eyes were harvested at day 30. **B**. Eyes were fixed and analyzed for H&E and immunohistochemistry for TUNEL to detect apoptotic cells. Results are representative of analysis from 5 mice in each group. **C**. High magnification of H&E and TUNEL staining.

We next determined if co-inhibiting the PI3K and mTOR pathways caused apoptosis of tumor cells in our retinoblastoma mouse model. This tumor model expresses Cre recombinase from the CHX10 promoter to inactivate RB1 and p107 genes in the retina to deregulate E2F-dependent proliferation and apoptosis [19]. Additional co-deletion of Pten blocks apoptosis, and these mice rapidly develop uniform bilateral retinoblastomas.. Without deletion of Pten, retinal cells quickly undergo E2F-dependent apoptosis until little of the retina remains, and tumorigenesis is consequently suppressed. We hypothesized that these tumors might undergo apoptosis triggered by inactivating the PI3K/Akt pathway. We administered vehicle or BEZ235 by oral gavage to our CHX-Cre^+^; Rb1^fl/fl^; p107^−/−^; Pten^fl/fl^ triple knockout (TKO) mice beginning on post-natal day 2 and given daily consecutively until day 6 and again between days 22-26 (Figure 4A). Eyes were harvested on day 30 for histology and TUNEL analysis and shown at low (4B) and high (4C) magnification. Vehicle treatment caused no detectable TUNEL^+^ apoptotic cell death. By contrast, BEZ235 treatment caused significant increases in the number of TUNEL^+^ cells and tumor cell clearance.

**Figure 5:**
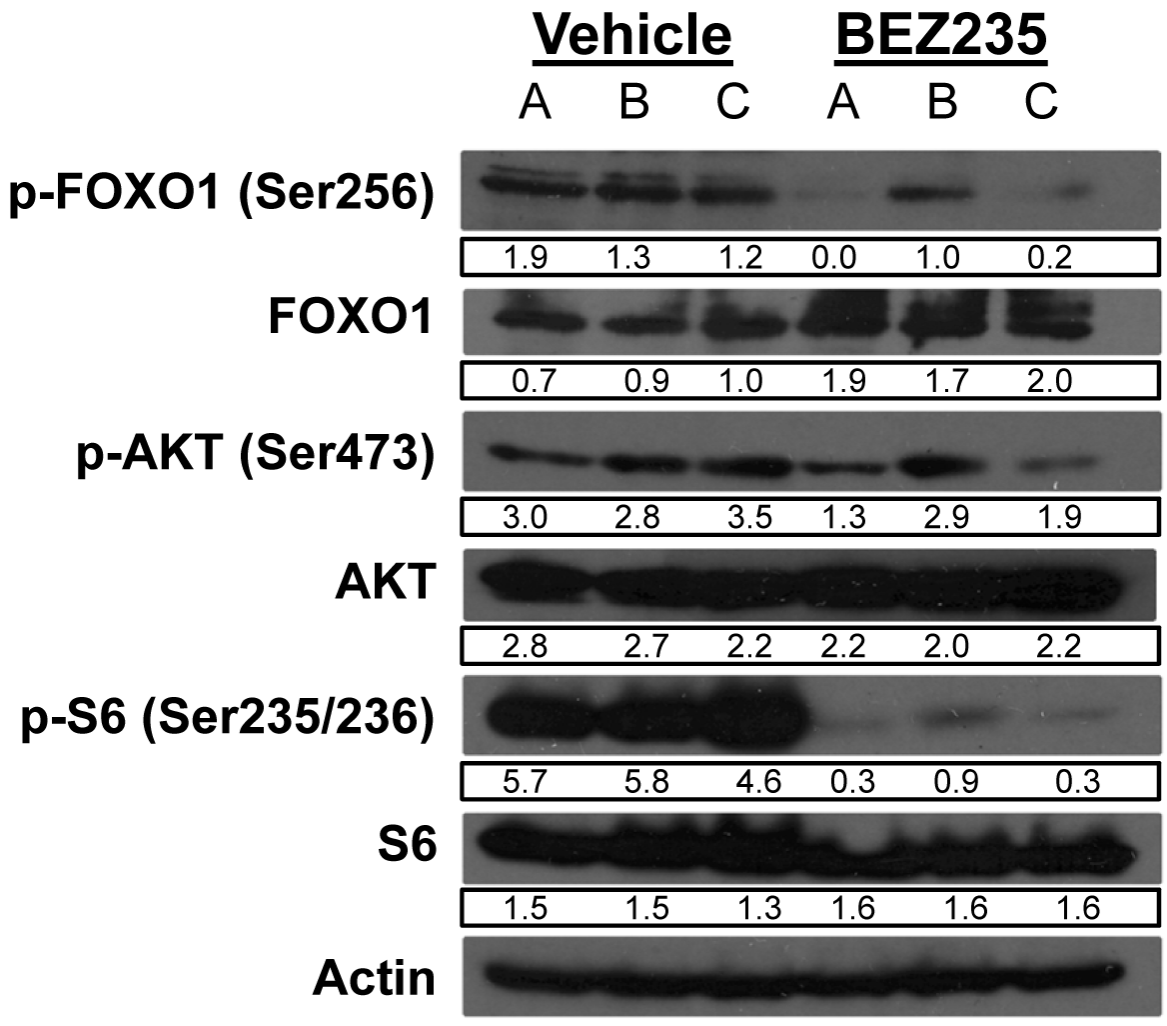
BEZ235 treatment suppresses FOXO1 and S6 phosphorylation in murine retinoblastoma Vehicle or BEZ235 was administered daily to *CHX-Cre^+^; Rb1^fl/fl^; p107^−/−^; Pten^fl/fl^* pups on post-natal days two through six, and retinae were harvested at day seven for immunoblot analysis using the listed antisera specific to p-FOXO1 (Ser256), FOXO1, p-AKT (Ser473), AKT, p-S6 (Ser235/236), S6, and ACTIN. A, B, & C are independent replicates from separately gavaged animals.

Because BEZ235 induced retinoblastoma tumor cell apoptosis, we tested how administration of BEZ235 affects signaling of the PI3K/Akt pathway components. Vehicle or BEZ235 was administered to TKO pups on post-natal days 2-6 and retinas were harvested at day 7 for immunoblot analysis using the listed antisera (Figure 5). A, B, & C are functional replicates from independently treated mice. P-FOXO levels are significantly reduced by treatment with BEZ235 compared to vehicle. Similar to effects in the Y79 and Weri1 cell lines, treatment with BEZ235 only modestly affected p-AKT levels in these Pten driven tumors, although 2 of 3 samples did show slightly reduced levels (lanes A & C). S6 is highly phosphorylated in the retina of TKO mice, and BEZ235 strongly reduced p-S6 levels in 3 of 3 mice. BEZ235 treatment did not affect levels of FOXO1, AKT1, S6 or ACTIN. These findings indicate that BEZ235 is able to cross the blood-retinal barrier and reduce p-FOXO1 and p-S6 levels, without stably reducing p-AKT levels and these results *in vivo* are highly similar to the *in vitro* results seen using the Y79 and Weri1 cell lines.

**Figure 6:**
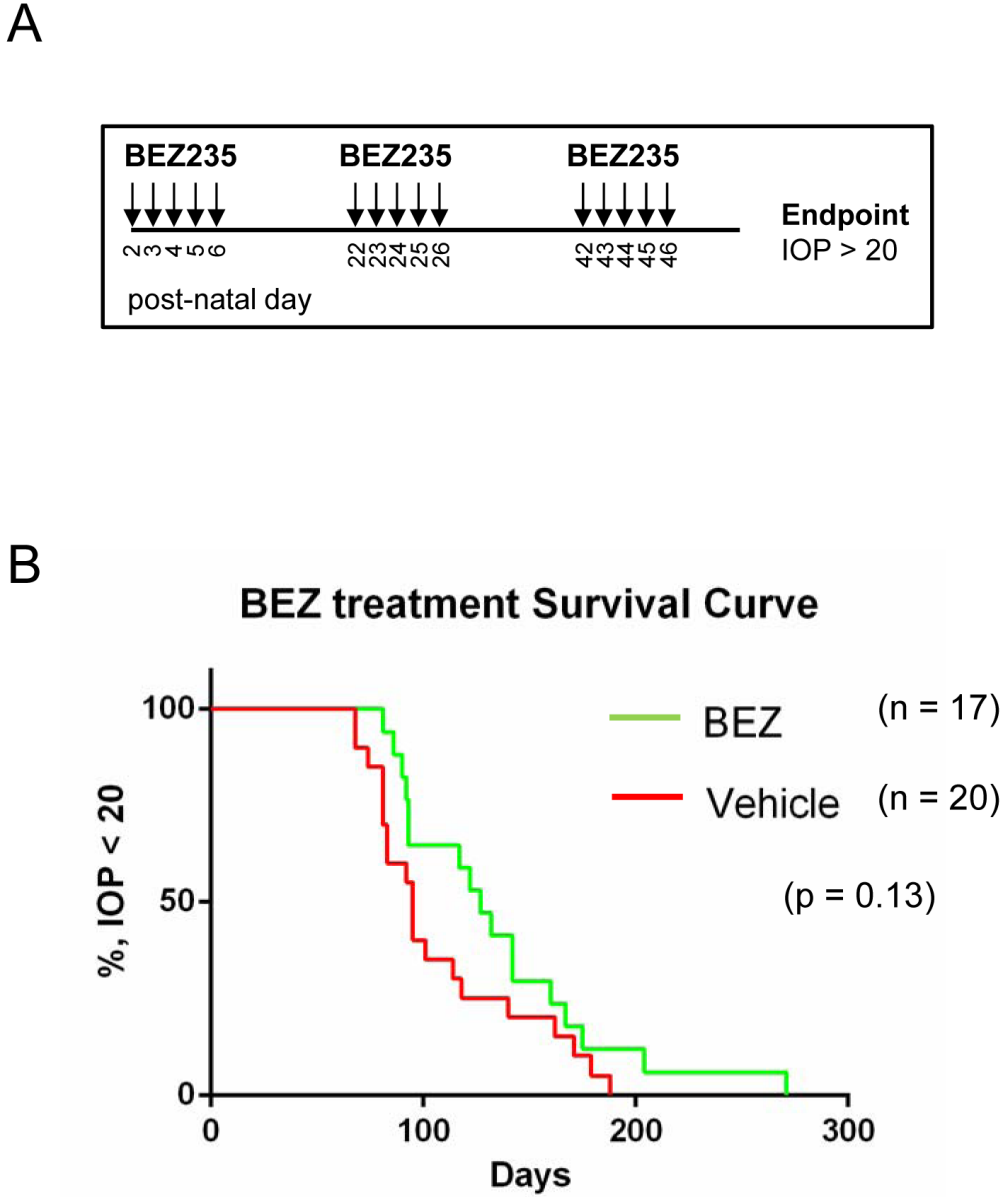
BEZ235 treatment does not significantly delay tumor-induced intraocular pressure of retinoblastoma bearing mice **A**. TKO mice were treated daily with vehicle or BEZ235 beginning at post-natal days 2 through 6, 22 through 26, 42 through 46, and intraocular pressure (IOP) was measured until an endpoint measurement of 20 mmHg was reached. **B**. Kaplan-Meier analysis of vehicle and BEZ235 treated retinoblastoma bearing mice.

Because BEZ235 effectively caused cell death in murine retinoblastomas, we determined if monotherapy treatment was able to delay intraocular pressure (IOP) caused by growing tumor burden. 20 TKO mice were sham treated with vehicle gavage and 17 mice were treated with a BEZ235 regimen according to the drug delivery schema shown in Figure 6A. Briefly, mice were administered vehicle or BEZ235 by oral gavage on post-natal days 2-6, 22-26, & 42-46 and IOP measured routinely. Once IOP reached 20 mm/Hg in a single eye it was considered an endpoint for Kaplan-Meier analysis (Figure 6B). Treatment with BEZ235 extended median survival from 95 days (vehicle) to 127 days (BEZ235), however comparison of survival curves was not significant (p = 0.13, Log-rank Mantel-Cox test; p = 0.07, Gehan-Breslow-Wilcoxon test).

**Figure 7:**
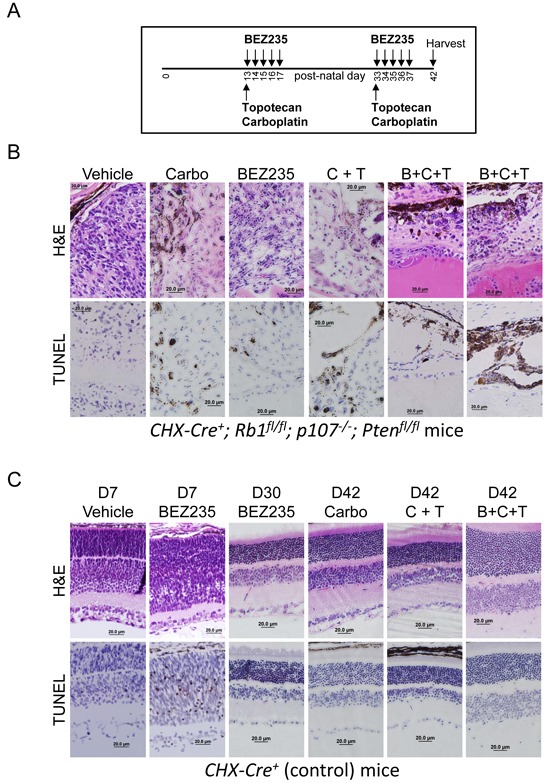
BEZ235 augments carboplatin and topotecan induced apoptosis of retinoblastoma tumor cells *in vivo* **A**. BEZ235 (50 μg/kg) was delivered by oral gavage on post-natal days 13 through 17, and 33 through 37. Topotecan (0.7 mg/kg) was delivered intraperitoneally on days 13 and 33. Carboplatin (100 μg/eye) was injected into the subconjunctival space on days 13 and 33. Mice were harvested at post-natal day 42 for analysis. **B**. *CHX-Cre^+^; Rb1^fl/fl^; p107^−/−^; Pten^fl/fl^* pups were treated with the noted therapy as described by the schema in A. Eyes were harvested at day 42, fixed and analyzed for H&E and immunohistochemistry for TUNEL to detect apoptotic cells. Carbo indicates monotherapy with carboplatin. C + T is combined treatment of carboplatin and topotecan. B + C + T is the combined treatment of all three, and results depict eyes from at least 3 independent mice per group. **C**. *CHX-Cre^+^* (control) pups were treated with the noted therapies. Vehicle or BEZ235 was administered daily to control mice beginning at post-natal day 2 and ending at day 7 when eyes were harvested for histological analysis (D7) to assess BEZ235-mediated apoptosis in the developing retina. BEZ235 was also given daily to control mice with mature retina beginning at day 25 until day 29 and eyes harvested at day 30 and analyzed for H&E and TUNEL to detect apoptotic cells (D30 BEZ235). The other listed therapies (Carbo, C+T, and B+C+T) were given to control mice as outlined by the schema in A and harvested for histology at day 42 from a minimum of three independent mice. Eyes were removed, fixed, and analyzed for H&E and TUNEL to detect apoptotic cells.

BEZ235 did not cause all tumor cells to undergo apoptosis or significantly extend the lifespan of the mice as a monotherapy. We therefore treated mice with BEZ235 in combination with the chemotherapeutic agents topotecan and carboplatin and measured the effects on tumor cell death. Vehicle and drugs were administered as outlined in the schema (Figure 7A). Treatment was begun on day 13 so that subconjunctival injections of carboplatin could be delivered after the mouse eyelid opened. BEZ235 was delivered daily by oral gavage on post-natal days 13 through 17 and 33 through 37. Topotecan was delivered intraperitoneally on days 13 and 33. Carboplatin was injected into the subconjunctival space on days 13 and 33. Eyes were harvested at day 42, fixed and analyzed for H&E and immunohistochemistry for TUNEL to detect apoptotic cells (Figure 7B). “Carbo” was monotherapy with carboplatin. “C + T” was combined treatment of carboplatin and topotecan, and “B+C+T” is the combined treatment of all three. Results are representative of at least three independent experiments. Combined treatment with BEZ235, topotecan and carboplatin caused significantly increased retinoblastoma cell apoptosis and tumor clearance than either treatment alone.

We noticed that BEZ235 mono-therapy was less effective causing apoptosis when administration was begun at day 13, compared to day 2 (Figure 4). To determine the potential cause, we tested if BEZ235 administration led to increased apoptosis in developing compared to mature retinal control cells. As per the schema used in Figure 4, Vehicle or BEZ235 was administered daily to 2-day old control mice for 5 consecutive days, and eyes were harvested at day seven for detection of apoptotic cells (Figure 7C). Unlike vehicle treatment, which caused no detectable apoptosis, treatment with BEZ235 resulted in retinal disorganization and significant TUNEL^+^ apoptotic cells. BEZ235 was also given daily to control mice with mature retina beginning at day 25 until day 29 and eyes harvested at day 30 and analyzed for H&E and TUNEL to detect apoptotic cells. Unlike developing retina, which underwent apoptosis during BEZ235 treatment, we detected no TUNEL^+^ cells in 30-day old differentiated retinas. These findings indicate that developing tissues, which may utilize PI3K-dependent pathways to control proliferation or differentiation, may be more susceptible to BEZ235-mediated apoptosis related tissue damage compared to effects on post-differentiated tissue. Carboplatin, carboplatin + topotecan (C+T), or combined treatment of BEZ235, carboplatin and topotecan (B+C+T) were delivered to control mice as outlined in the schema in 7A and harvested at post-natal day 42. Importantly, none of these treatments, even combined treatment with all three, caused apoptosis in control retinal cells (Figure 7C).

## DISCUSSION

Retinoblastoma tumors typically develop in infants and children during the process of retinal development when trophic factors are present to direct differentiation and block cell death caused by loss of *RB1* [25–27]. It is hypothesized that retinoblastoma tumors develop with so few other mutations besides *RB1* itself owing to a unique cell-intrinsic signaling circuitry [15, 16]. For example, this circuitry elevates levels of the MDM2 oncogene to minimize p53-mediated apoptosis [16, 18]. Likewise, the S-phase associated kinase 2 (SKP2) regulate cell cycle entry and is required for cone precursor and retinoblastoma cell proliferation [16, 28, 29]. Both MDM2 and SKP2 are themselves targets of AKT phosphorylation mediated activation, suggesting that activated AKT may also be part of this circuitry [30–33]. We show that AKT is activated in human retinoblastoma in this study. The most common mutation types activating the AKT pathway in other cancer types are inactivation of *PTEN* tumor suppressor gene, which is not mutated in human retinoblastoma, or gain-of-function point mutations in PI3K. *A*ctivating mutations in *PIK3CA* have only rarely been observed in human retinoblastoma [34]. The spleen associated tyrosine kinase (SYK) is widely overexpressed in human retinoblastoma because of altered epigenetic regulation, and this kinase activates AKT in diffuse large B-cell lymphomas, but their relationship in retinoblastoma is unclear [7, 35]. The microRNA miR-17-92 (*MIRC1*) cluster activates AKT in lymphoproliferative disease and is amplified in human retinoblastoma and promotes tumors by reducing apoptosis [36–38].

Once activated, AKT phosphorylates multiple targets that regulate proliferation, apoptosis, metabolism, and protein synthesis [20]. FOXO1, a direct target of AKT, is highly phosphorylated and thus inactivated in human retinoblastoma tumors that also had high p-AKT levels. AKT can activate mTOR by phosphorylating TSC2 and relieving its inhibition of mTOR [39]. We do not observe strong activation of the ribosomal S6 protein, which is downstream of mTOR, in retinoblastoma. It is unclear why AKT targets p-FOXO1 more strongly than p-S6 in human retinoblastoma. We previously developed a murine model of retinoblastoma that deleted RB1 and p107 to unleash E2F-driven proliferation and apoptosis, and co-deleted Pten to suppress apoptosis and form bilateral retinoblastoma tumors [19]. We do observe p-AKT activation in wild-type and ΔRb/p107 retinae. But we do not observe rapid tumor emergence in the ΔRb/p107 retinae, only late onset unilateral tumors. This suggests that “normal” levels of AKT activity is insufficient to offset the strong pro-apoptotic stimulus from deregulated E2F1 following loss of both Rb and p107 in mice, which do not develop retinoblastoma from Rb deficiency alone [40, 41]. Only by co-deleting *Pten*, or overexpressing hyperactive forms of PIK3CA or AKT, or a dominant-negative form of FOXO1, suppresses apoptosis and induce tumors. Together, these data indicate that it is rational to target the PI3K/Akt pathway in human retinoblastoma [19].

Widespread activation of the PI3K/Akt/mTOR pathway in human cancer has made it an attractive target for therapeutic inhibition. NVP-BEZ235 is among a second generation of PI3K inhibitors with increased specificity, reduced toxicity, and fewer off-targets [42]. Studies in various cancer cells indicated that cell lines with PI3K hotspot mutations are more susceptible to treatment with PI3K inhibitors than those with *Pten* mutations [43]. In human patients, PIK3CA mutant breast tumors acquire mutations in *Pten* following treatment with BEZ235 in the clinic, which likely further activates AKT [44]. A number of these cell lines tested only underwent growth arrest but not apoptosis when treated with 10 μM BEZ235 for 72 hours [43]. Results from our study indicate that Y79 fits into this BEZ235 resistant category, and Weri1 falling somewhere between Y79 and the highly sensitive cell lines reported previously [42]. BEZ235 crossed the blood-retinal barrier and caused cell apoptosis in our ΔRb/p107/Pten retinoblastoma mouse model. This monotherapy-based induction of apoptosis did not lead to a significant delay in tumor burden, perhaps in part because our BEZ235 dosing regimen did not recapitulate the daily dosing schedule that a pediatric population would likely receive. BEZ235 treatment only led to modest inhibition of p-AKT levels, but it was much stronger at reducing levels of both p-S6 and p-FOXO1 *in vitro* and *in vivo*. It is unclear why p-FOXO1 levels were reduced without correlating reduction in p-AKT, but inhibition of the PI3K and AKT pathways is widely complicated by multiple feedback regulatory loops [45–49]. In particular, FOXO-mediated feedback following treatment with BEZ235 causes gene expression of growth factor receptors that further stimulate p-AKT [50].

We observed significant toxicity of BEZ235 in newborn mice. Previous studies delivered 40 mg/kg BEZ235 to adult mice [42]. In our studies, 50 μg/kg BEZ235 was toxic to newborn (post-natal day 0) mice, whereas post-natal day 2 pups tolerated this concentration. It should also be noted that normal, developing retinae were susceptible to BEZ235-induced apoptosis, whereas fully mature retina did not undergo similar cell death. This indicates that retinal cells likely utilize the PI3K/Akt signaling pathway during normal development, which when perturbed by PI3K inhibition causes cell death. These findings raise concern about the use of BEZ235 in infants with still-developing tissue. Mice and humans age differently in distinct developmental stages [51]. During the weaning period when both species develop retinoblastoma tumors, it was calculated that one mouse day is roughly equivalent to 6.43 human days. The toxicity of BEZ235 we observe at post-natal day 7 in the retina of mice correlates to an age of approximately 45 human days. In contrast, BEZ235 treatment beginning at mouse post-natal day 13, or roughly 3 human months old, did not induce apoptosis. Several adult clinical trials involving BEZ235 listed atClinicalTrials.gov have been discontinued due to poor tolerability or modest anti-tumor activity [52, 53]. Because of the apparent limitations of treatment with BEZ235 monotherapy, we considered if efficacy could be improved by combining with chemotherapeutics against retinoblastoma cells. Co-administration of BEZ235 with topotecan/carboplatin significantly increased apoptosis and tumor cell clearance over that of either treatment alone. Importantly, co-therapy with BEZ235, topotecan and carboplatin doesn't induce apoptosis in non-malignant mature retinal tissue. BEZ235 systemic toxicity could also potentially be minimized by delivering it as localized therapy directly to the eye, as has been demonstrated with other small molecule inhibitors such as Mdm2 or Syk of other signaling pathways activated in retinoblastoma [2, 7]. Our study has increased the variety of potentially effective targeted treatments that can be considered for human retinoblastoma.

## MATERIALS AND METHODS

### Experimental animals

The *Chx10-Cre; Rb^Lox/Lox^; p107*^−/−^*; Pten^Lox/Lox^* mouse model of retinoblastoma and related PCR genotyping protocols have been described [19]. We obtained these mice from The Jackson Laboratory and bred them together and used recommended PCR primers to genotype. All mouse experiments were performed in accordance with University of Minnesota Institutional Animal Care and Use Committee procedures and guidelines. NVP-BEZ235 (Fisher Scientific; #NC0452160) was delivered to mice 50 μg/kg by oral gavage. Topotecan (0.7 mg/kg) was given by IP injection and Carboplatin (100 ug/eye) by subconjunctival injection. A rodent tonometer (Tonolab) was used to measure the intraocular pressure (IOP) of sedated mice by averaging six independent measurements. Kaplan-Meier curves were calculated using GraphPad Prism software.

### Retinoblastoma immunohistochemistry

Retinoblastoma tissue microarrays were purchased from US Biomax (#BC35111a). IHC was performed as previously described [19, 54]. Briefly, detection was through primary antibodies against p-AKT1, p-FOXO1, p-S6 & Ki-67 (SP6) (Biocare Medical, Cat# CRM325), using Vector biotinylated secondary (1:250), and streptavidin-horseradish peroxidase as tertiary (Covance #SIG-32000) and chromagen 3,3′-diaminobenzidine substrate (Covance #SIG-31043). TUNEL IHC analysis used the ApopTag Peroxidase kit from EMD Millipore (S7100). The University of Minnesota BioNet Resource assisted with H&E staining, KI-67 & TUNEL and other IHC staining as previously described [55]. All antisera used for IHC and immunoblotting are listed in Supplementary Table S2. Fisher's exact test was used to calculate two-tailed P-values for the contingency table comparisons.

### Cell culture and protein immunoblotting

Y79 (HTB-18) and WERI-Rb-1 (HTB-169) cell lines were obtained directly from ATCC and were cultured in RPMI1640 media containing 20% FBS. Immunoblotting was performed as described [11]. Cell viability was measured with a Countess automated cell counter and graphed as the percentage of viable cells compared to total cells for each time point as previously described [56]. Caspase-3 apoptosis detection was performed using a kit from BD Biosciences (#550914) following manufacturer instructions and as previously described [57]. P-values were determined by Student's t-test to determine of two sets of data are significantly different from each other. ImageJ software was used to quantify band intensities from immunoblots. Numbers are presented under each band and are only comparable to each other within the same gel.

## SUPPLEMENTARY TABLES




